# Supplementary material for: In Situ Raman Microdroplet Spectroelectrochemical Investigation of CuSCN Electrodeposited on Different Substrates
Source: Nanomaterials (Basel). 2021 May 11;11(5):1256. doi: 10.3390/nano11051256 (PMC8151883; doi:10.3390/nano11051256)
Supplement: Supplementary file 1 [file nanomaterials-11-01256-s001.zip › nanomaterials-1179538-supplementary.pdf]

nanomaterials

Supporting Information

In situ Raman Microdroplet Spectroelectrochemical Investigation of CuSCN Electrodeposited on Different Substrates

Zuzana Vlčková Živcová *, Milan Bouša, Matěj Velický, Otakar Frank and Ladislav Kavan

J. Heyrovský Institute of Physical Chemistry, Czech Academy of Sciences, Dolejškova 2155/3, 182 23 Prague, Czech Republic; milan.bousa@jh-inst.cas.cz (M.B.); matej.velicky@jh-inst.cas.cz (M.V.); otakar.frank@jh-inst.cas.cz (O.F.); ladislav.kavan@jh-inst.cas.cz (L.K.)

* Correspondence: zuzana.vlckova@jh-inst.cas.cz

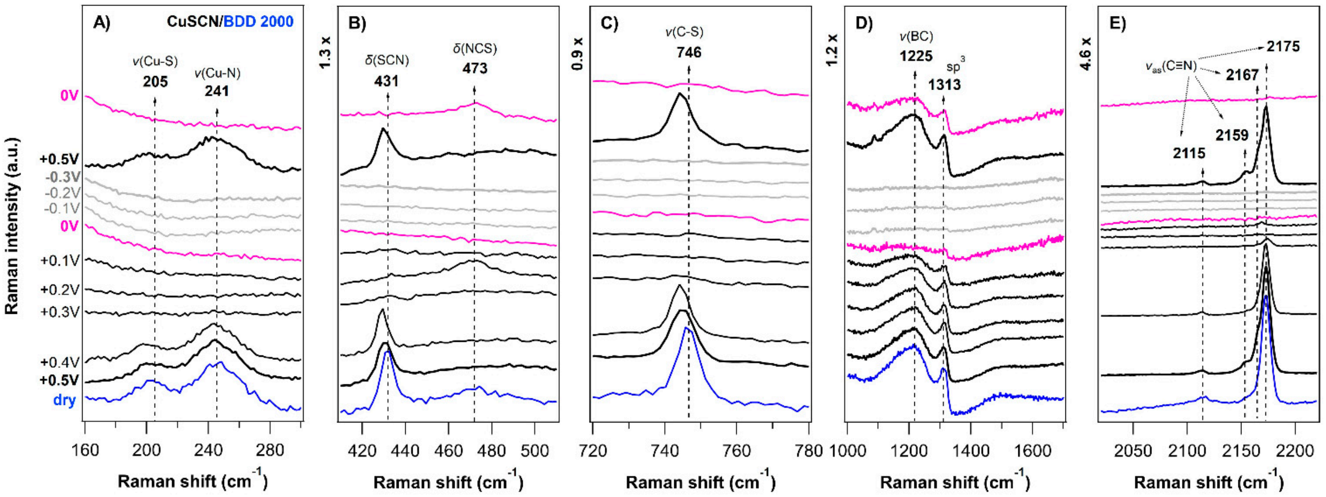

Figure S1. In situ Raman-μSEC spectra of the CuSCN layer (spectral regions of 160–300, 410–510, 720–780, and 2020–2220 cm^{-1} in charts **A**, **B**, **C**, and **E**, respectively) electrodeposited on the BDD 2000 substrate (spectral region of 1000–1700 cm^{-1} in chart **D**) with B/C ratio in the gas phase 2000 ppm as a function of the applied potential (vs. Ag/AgCl; shown on the left-side chart) in 6 M LiCl. The measurement sequence is as follows: the first spectrum was acquired at +0.5 V (bottom thick black trace), and the last spectrum was acquired at 0 V (top thick pink trace). The sequence of negative potentials (from −0.3 V to −0.1 V) is visualized by grey traces and the sequence of positive potentials (from +0.1 V to +0.5 V) is visualized by black traces. The reference (ex situ) Raman spectrum of dry CuSCN layer on BDD 2000 substrate is shown for comparison (blue trace). Spectra are offset for clarity; the intensity scale is identical for all the charts.

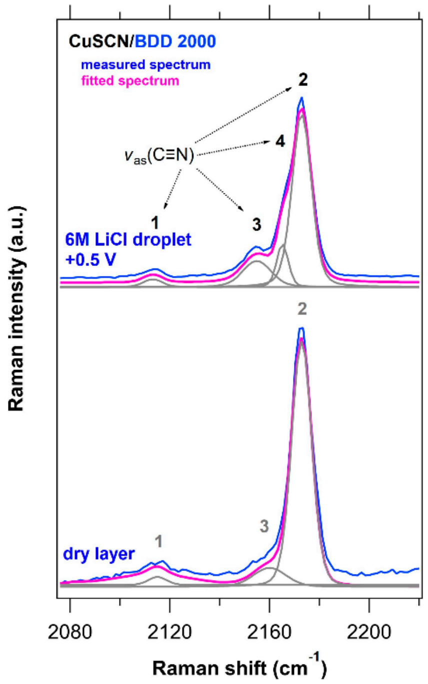

Figure S2. Raman spectra (blue trace) of electrodeposited CuSCN i) dry (ex situ) layer (bottom), and ii) in 6 M LiCl microdroplet (top) at +0.5 V vs. Ag/AgCl potential upon the in situ Raman-µSEC measurement, on the BDD 2000 substrate in the region of SCN vibrations (2080–2200 cm^{-1}). The pink and gray traces represent the fits and individual peak positions, respectively, using Voigt lineshapes.

Table S1. Fitting parameters of Raman ν(C–S) position in the CuSCN layers electrodeposited on various substrates (GC, BDD 1000 and 2000 ppm, HOPG, FTO); i) dry (ex situ) layer, and ii) in 6 M LiCl microdroplet at +0.5 V vs. Ag/AgCl potential upon the in situ Raman-µSEC measurement.

		**Peak1**	
		Raman shift	FWHM
		(cm^{-1})	
CuSCN/GC	**dry layer**	746.3	*8.2*
	**+0.5 V** µ-droplet	747.7	*8.8*
CuSCN/BDD	**dry layer**	745.1	*8.2*
	**+0.5 V** µ-droplet	743.8	*6.6*
CuSCN/BDD2000	**dry layer**	746.5	*6.9*
	**+0.5 V** µ-droplet	744.5	*6.9*
CuSCN/HOPG	**dry layer**	746.8	*8.5*
	**+0.5 V** µ-droplet	746.8	*3.7*
CuSCN/FTO	**dry layer**	745.8	*10.8*
	**+0.5 V** µ-droplet	744.3	*7.9*

Table S2. Fitting parameters of Raman ν_{as}(C≡N) position in the CuSCN layers electrodeposited on various substrates (GC, BDD 1000 and 2000 ppm, HOPG, FTO); i) dry (ex situ) layer, and ii) in 6 M LiCl droplet at +0.5 V vs. Ag/AgCl potential upon the in situ Raman-µSEC measurement.

		Peak1		Peak2		Peak3		Peak4	
		Raman shift	*FWHM*	Raman shift	*FWHM*	Raman shift	*FWHM*	Raman shift	*FWHM*
		**(cm⁻¹)**		**(cm⁻¹)**		(cm⁻¹)		(cm⁻¹)	
**CuSCN/GC**	**dry layer**	**2114.7**	***13.4***	**2174.5**	*10.6*	2158.3	*23.9*	–	–
	**+0.5 V** µ-droplet	2116.3	*12.5*	2176.8	*9.2*	2169.1/*21.2**			
CuSCN/BDD	**dry layer**	2114.1	*10.3*	2173.7	*9.4*	2165.9/*23.0**			
	**+0.5 V** µ-droplet	2111.8	*8.2*	2172.5	*8.6*	2158.0	*17.5*	2165.3	*5.1*
CuSCN/BDD2000	**dry layer**	2114.1	*35.8*	2172.9	*9.1*	2165.8	*25.7*	–	–
	**+0.5 V** µ-droplet	2113.1	*9.1*	2172.9	*9.1*	2154.9	*13.4*	2165.1	*5.5*
CuSCN/HOPG	**dry layer**	2115.1	*10.6*	2174.9	*10.3*	2159.1	*24.4*	–	–
	**+0.5 V** µ-droplet	2115.6	*7.5*	2174.5	*6.5*	2159.4	*6.4*	–	–
CuSCN/FTO	**dry layer**	2114.5	*12.2*	2174.6	*13.6*	2159.2	*17.2*	2167.1	*5.1*
	**+0.5 V** µ-droplet	2111.7	*10.5*	2175.7	*6.7*	2156.1	*12.3*	2166.5	*5.6*

* due to the low intensity of the shoulder bands, the deconvolution into two peaks (3, 4) was not reliable, and a single peak was used instead

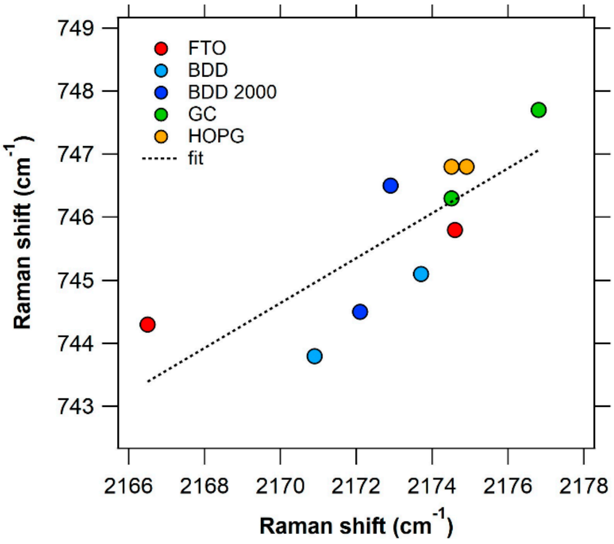

Figure S3. Correlation between the Raman shifts of the C−S stretching vibration at ~745 cm^{-1} and the C−N stretching vibration at ~2175 cm^{-1} (fitted as a single Voigt lineshape each). The dotted line represents a least-squares linear fit; the Pearson's correlation coefficient is 0.8.
